# Satiety differentially modulates feeding steps in the jellyfish *Cladonema*

**DOI:** 10.1016/j.isci.2025.112192

**Published:** 2025-03-10

**Authors:** Genta Mashiba, Hiromu Tanimoto, Vladimiros Thoma

**Affiliations:** 1 Graduate School of Life Sciences, Tohoku University, Katahira 2-1-1, Sendai 980-8577, Japan

**Keywords:** Biological sciences, Zoology, Ethology

## Abstract

Following a meal, animals exhibit satiety, a state of decreased motivation to feed. Satiety is observed throughout the animal kingdom, suggesting ancient underlying mechanisms. Here, we investigate how satiety alters feeding in jellyfish, species that lack a centralized brain. Using comprehensive ethological analyses in *Cladonema*, a jellyfish with highly stereotyped, sequential feeding behavior, we show that satiety disorganizes its feeding sequence and delays all feeding steps, thus reducing food consumption. Surprisingly, isolated tentacles from fed jellyfish displayed satiety, thereby showing sustained and autonomous signaling of this state. Moreover, temporal dynamics of inhibition differed among feeding steps. Taken together, our results highlight complex satiety signaling in this species, suggesting multiple underlying signals.

## Highlights

• In hungry *Cladonema* jellyfish, feeding is a well-organized sequence of steps
• *Ad libitum* feeding satiates the jellyfish, disorganizing and delaying these steps
• Satiety inhibits the feeding steps with distinct temporal dynamics

## Graphical abstract

**Figure:**
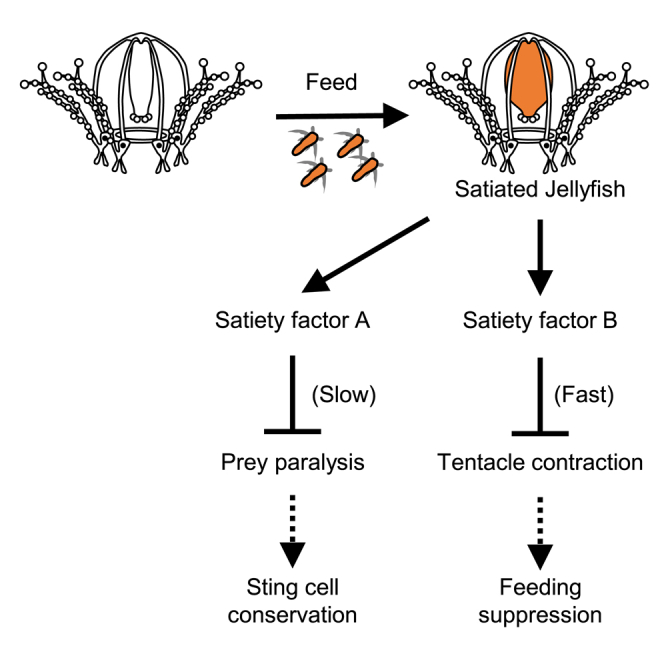

## Introduction

Feeding regulation is necessary for energy homeostasis in animals. Avoiding over-eating is achieved through satiety: the decreased motivation to feed that follows a meal. Satiety can suppress consumption by inhibiting any step or aspect of the behavioral sequences of foraging or ingestion.^1^ Moreover, it regulates additional behavioral traits, inhibiting locomotion and promoting sleep. Such effects occur in many diverse species that include rodents,^2^^,^^3^ pigeons,^4^ crayfish,^5^ fruit flies,^3^^,^^6^^,^^7^ nematodes^8^ and even the freshwater polyp *Hydra*.^9^ In the fruit fly *Drosophila*, modulation of sensory and central neurons is critical for state-dependent regulation of feeding behaviors.^10^^,^^11^ Modulatory molecules form a complex network of satiety signals.^12^ Interestingly, some feeding-regulating neuropeptides are conserved across evolutionarily distant species, such as GLWamide/myoinhibitory peptide^13^ and neuropeptides F/Y.^14^ The presence of multiple satiety signals is likely necessary to regulate multiple behavioral traits. One of the general functions of the central nervous system (CNS) is to coordinate such signals, for example through hierarchically organized hormone and neuropeptide pathways. However, how these complex systems arose and evolved remains unclear.

To approach this question, we focused on jellyfish. Jellyfish belong to the phylum cnidaria, the sister group of bilaterians, and have decentralized nerve nets^15^ that may represent the elementary nervous systems of their ancestors.^16^^,^^17^ Nevertheless, they show well-organized feeding that includes foraging and ingestive behaviors, as in other species.^18^ To forage, jellyfish employ strategies that include ambush predation, trawling,^19^ filter-feeding,^20^ and aggressive mimicry.^21^ In contrast to these diverse foraging strategies, ingestive behaviors are more uniform and involve capturing prey and consuming it. Satiety could exert its effects on any of these processes.

The first step of cnidarian feeding is sting cell (nematocyte)-mediated prey capture. This step is crucial, as jellyfish strains that lack sting cells are feeding-deficient.^22^ Inhibition of prey capture is the most well documented effect of satiety across several cnidarians.^13^^,^^23^^,^^24^^,^^25^^,^^26^^,^^27^^,^^28^^,^^29^ Since nematocytes are single-use cells, reduced capture efficiencies may be due to nematocyte reduction after feeding, increased thresholds of nematocyst discharge, or both.

Following prey capture, cnidarians execute coordinated tentacle and mouth movements^30^^,^^31^ to ingest it. Satiety also influences these feeding steps,^9^^,^^13^^,^^31^^,^^32^^,^^33^ but it is unclear if it modulates their thresholds and/or motor actions. Moreover, the most crucial feeding step for explaining reduced consumption under satiety (if any) remains unknown.

Here, we use the jellyfish *Cladonema* to address these questions. *Cladonema* are semi-sessile: they can attach themselves to a substrate via adhesive tentacle branches, making them advantageous for detailed feeding behavior analysis. We offered jellyfish single brine shrimps (prey), recorded their behavior, and carried out ethological analyses to comprehensively describe and quantify their feeding. Our results show that in jellyfish, satiety differentially modulates multiple steps of feeding, which jointly contribute toward suppressing food consumption.

## Results

### Feeding in starved and fed jellyfish

**Figure 1:**
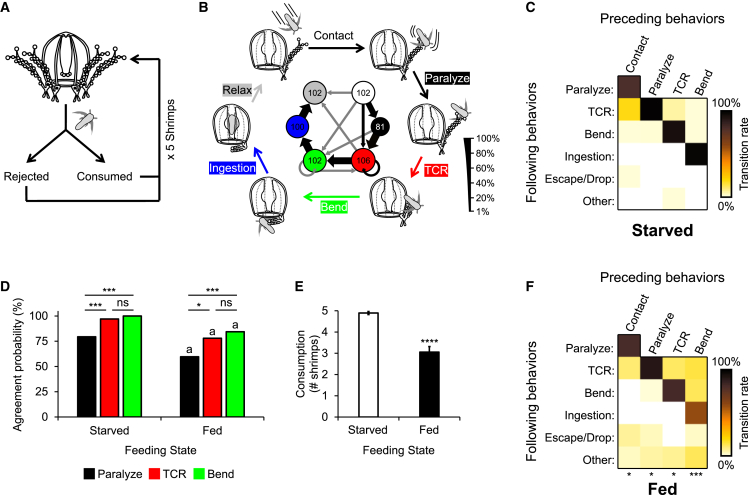
The feeding sequence in starved and fed *Cladonema* jellyfish (A) Schematic of the experiment used to analyze *Cladonema* feeding sequence. (B) Schematic of feeding steps and diagram of step transitions in starved animals (center). Node arrow width represents transition probability. Transition probabilities below 5% are shown in gray. Node radius represents number of observations, as provided in each node. (C) Heat maps of transitional probabilities between feeding steps in starved animals. From left to right, *n* = 102, 81, 106, and 102 observations per heatmap. (D) Agreement probabilities between tentacle-mediated feeding steps (paralyze, TCR, and bend) and ingestion in starved and fed jellyfish (Fisher’s exact test with Bonferroni correction; ∗*p* < 0.05; ∗∗∗*p* < 0.001; ns *p* > 0.05). Comparisons between starved and fed animals are also indicated (Fisher’s exact test with Bonferroni correction; a *p* < 0.05). *n* = 102 (starved) or 109 (fed) observations. (E) Number of brine shrimps consumed for starved and fed jellyfish. Data are represented as mean ± SEM (Mann-Whitney test; ∗∗∗∗*p* < 0.0001). *n* = 19 (starved) or 18 (fed) jellyfish. (F) Heat maps of transitional probabilities between feeding steps in fed animals. From left to right, *n* = 109, 88, 120, and 113 observations per heatmap. Statistically significant differences from starved animals are indicated (Fisher’s exact test; ∗*p* < 0.05; ∗∗∗*p* < 0.001).

To characterize *Cladonema* feeding, we offered single brine shrimps (prey) to starved jellyfish (Figure 1A). We allowed them to capture and consume prey and video-recorded their entire feeding sequence. Starved animals showed stereotyped, sequential feeding behavior (Figures 1B and 1C). Relaxed jellyfish rapidly paralyzed prey when it contacted with their outstretched tentacles. Subsequently, jellyfish retrieved the prey by contracting the tentacle (tentacle contraction reflex [TCR]) followed by bending the contracted tentacle to bring the prey under the umbrella. The manubrium oriented toward the bent tentacle, ingested the prey, and the tentacle relaxed to its initial position (Figure 1B). We asked if occurrence or failure of each tentacle-mediated feeding step (paralyze, TCR, and bend) coincided with occurrence or failure of ingestion, respectively. To this end, we calculated the agreement probability between ingestion and each of these behaviors. This analysis showed that all steps had high agreement with ingestion, but TCR and bending were superior to prey paralysis, approaching 100% (Figure 1D, left). Taken together, these results show that starved jellyfish show a well-organized feeding sequence.

To understand how satiety affects the feeding sequence, we allowed jellyfish to feed *ad libitum* immediately prior to measurements. Feeding reduced consumption (Figure 1E), transitional probabilities of the feeding steps (Figure 1F) and agreement probabilities between tentacle-mediated steps and ingestion (Figure 1D, right). These results suggest a less goal-oriented state in fed jellyfish, as in fed *Drosophila*.^7^ Therefore, satiety disorganizes the feeding sequence in jellyfish.

**Figure 2:**
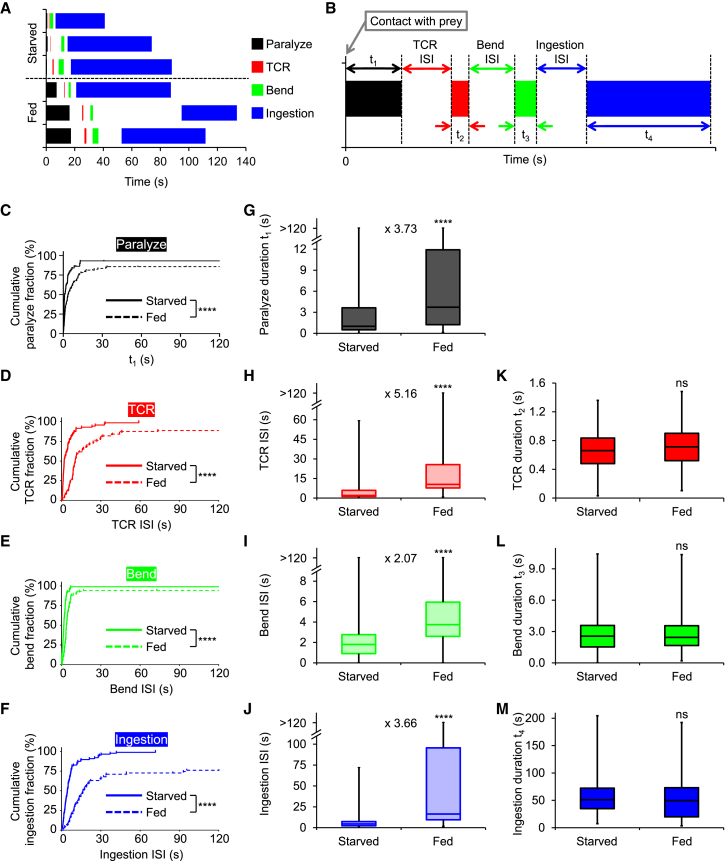
Satiety prolongs prey paralysis duration and inter-step intervals of downstream feeding steps (A) Examples of ethograms of feeding in starved and fed jellyfish. Contact with prey was defined as t = 0 s. (B) Idealized ethogram of feeding steps showing prey paralysis, tentacle contraction reflex (TCR), tentacle bending (bend) and ingestion. Inter-step intervals (ISIs) and step durations (t_1_–t_4_) are indicated. (C–F) Kaplan-Meier curves for cumulative fractions of paralyze (C), TCR (D), bend (E), and ingestion (F) for starved (continuous) and fed (dashed) animals (log rank (Mantel-Cox) test; ∗∗∗∗*p* < 0.0001). From top to bottom, *n* = 101, 79, 100, 87 and *n* = 108, 90, 104, 66 observations for starved and fed animals, respectively. (G–J) Effect of feeding on paralyze duration (G) and TCR, bend, and ingestion ISIs (H–J) (Mann-Whitney test; ∗∗∗∗*p* < 0.0001). Lower, intermediate and upper bounds of boxes represent T_25_s, T_50_s, and T_75_s, respectively. Whiskers show minima/maxima. Numbers between boxes indicate fold change of median (T_50_) duration (G) or ISI (H–J) induced by feeding. From top to bottom, *n* = 81, 78, 100, 87 and *n* = 100, 89, 103, 63 observations for starved and fed animals, respectively. (K–M) Durations of successfully executed TCR (K), bend (L), and ingestion (M) in starved and fed animals (Mann-Whitney test; ns *p* > 0.05). Lower, intermediate and upper bounds of boxes represent first, second and third quartiles, respectively. Whiskers show minima/maxima. From top to bottom, *n* = 105, 99, 82 and *n* = 118, 113, 38 observations for starved and fed animals, respectively.

Does satiety prolong latencies or durations of feeding steps? To address this question, we generated ethograms (Figure 2A) and quantified step durations and inter-step intervals (ISIs) for all feeding steps (Figure 2B). This analysis showed that satiety significantly prolonged prey paralysis duration (Figures 2C and 2G). Moreover, satiety had similar effects on dynamics of step transitions (Figures 2D–2F), increasing median ISIs (T_50_s) from 2- to more than 5-fold, with the greatest effect observed for TCR (Figures 2H–2J). However, satiety had no effect on the durations of TCR, bending or ingestion, when these steps were successfully executed (Figures 2K–2M). These results are in line with the impairments of transition rates (Figures 1C and 1F), leading to the conclusion that satiety reduces food intake by decreasing the probability of behavioral transitions. Moreover, prolongation of ISIs but not durations indicates impaired initiation of feeding steps but intact overall motor programs. Interestingly, there was no correlation between durations/ISIs of adjacent feeding steps (Figure S1), suggesting that they were independently controlled.

**Figure 3:**
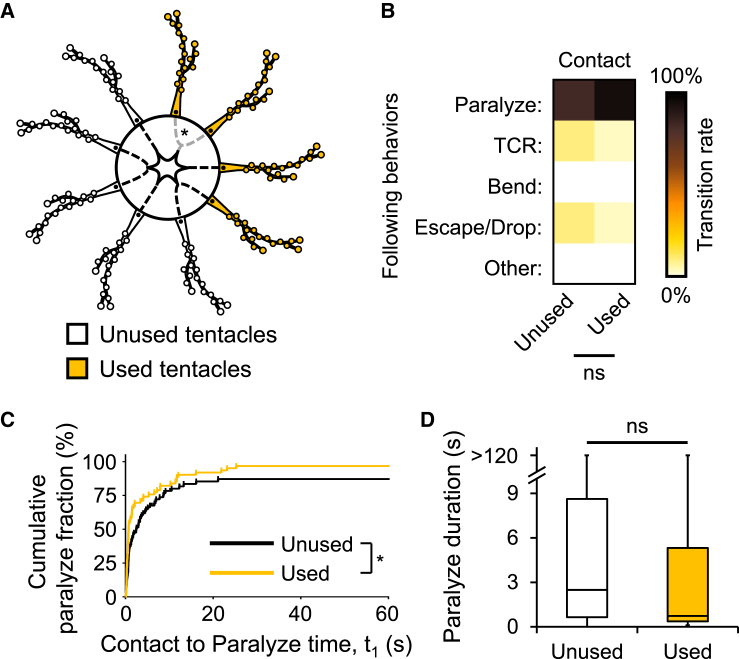
Tentacles previously used for feeding are unimpaired for paralyzing prey (A) Experiment schematic. Anesthetized shrimps were applied to only four tentacles (orange, “used”). Branched radial canals (asterisk) or other landmarks were used for tentacle tracking. Adhesive branches are omitted for simplicity. (B) Heat maps of transitional probabilities from prey contact to various following behaviors for used and unused tentacles (Fisher’s exact test; ns *p* = 0.1020). *n* = 79 and 69 observations for unused and used tentacles, respectively. (C) Kaplan-Meier curves for cumulative fractions of brine shrimp paralysis for observations with unused (black) and used (orange) tentacles (log rank (Mantel-Cox) test; ∗*p* = 0.0224). *n* = 79 and 69 observations for unused and used tentacles, respectively. (D) Median duration of prey paralysis following contact with previously unused (white) and used (orange) tentacles (Mann-Whitney test; ns *p* = 0.0564). Lower, intermediate and upper bounds of boxes represent T_25_s, T_50_s, and T_75_s, respectively. Whiskers show minima/maxima. *n* = 72, 67.

Nematocytes are single-use cells that cnidarians must renew. Therefore, sting cell reduction could cause the impairments in prey paralysis observed in satiated jellyfish (Figures 1 and 2). To test this possibility, we fed jellyfish with an abundance of anesthetized prey, targeting only four of their tentacles (“used” tentacles; Figure 3A). We then offered non-anesthetized prey and compared durations of prey paralysis and feeding behaviors between used and unused tentacles. “Used” tentacles were unimpaired (Figures 3B–3D). Thus, sting cell reduction due to previous use is not sufficient for prey paralysis inhibition. Unexpectedly, “used” tentacles were marginally but significantly more effective in capturing prey (Figure 3C), perhaps due to sensitization, which has been reported in cnidarians.^34^

### The manubrium is not necessary for maintenance of satiety in tentacles

**Figure 4:**
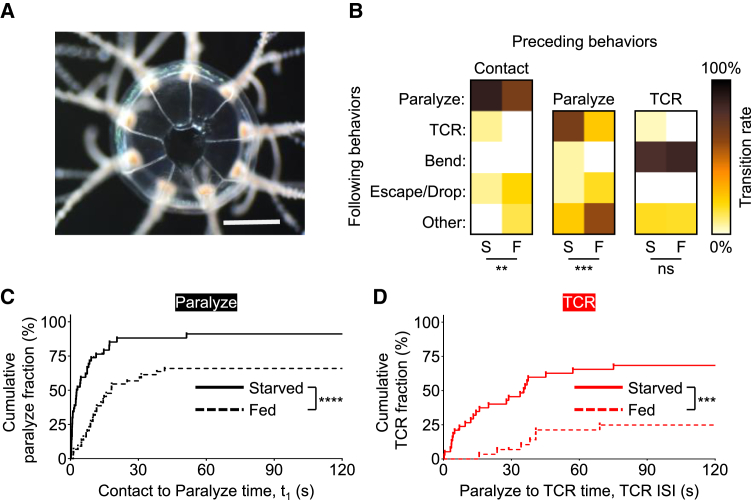
The manubrium is not necessary for maintenance of satiety in tentacles (A) Representative photograph of the bell of a jellyfish with resected manubrium. Scale bar, 1 mm. (B) Heat maps of transitional probabilities between feeding steps in starved (S) and fed (F) manubrium-less animals. From left to right, *n* = 44, 44, 38, 29, 29, and 6 observations per heatmap. Statistically significant differences from starved animals are indicated (Fisher’s exact test; ∗∗*p* = 0.0014; ∗∗∗*p* = 0.0009; ns *p* > 0.05). (C and D) Kaplan-Meier curves for cumulative fractions of paralyze (C) and TCR (D) for starved (continuous) and fed (dashed) manubrium-less animals (log rank (Mantel-Cox) test; ∗∗∗∗*p* < 0.0001; ∗∗∗*p* < 0.001). From left to right, *n* = 44, 38 and *n* = 44, 29 observations for starved and fed animals, respectively.

To better understand the mechanisms underlying satiety, we sought to identify the body parts required for it. Satiety signals likely originate in the manubrium, where ingestion and digestion take place. We therefore resected the manubrium in starved and fed jellyfish, following 1 h of *ad libitum* feeding on brine shrimps for the latter group. Manubrium-less jellyfish (Figure 4A) could survive and execute all tentacle-mediated steps of feeding. Indeed, the majority of manubrium-less starved animals captured and paralyzed prey, and also contracted and bent their tentacles (Figures 4B–4D). Surprisingly, both prey paralysis and TCR were suppressed in freshly fed jellyfish despite the absence of the manubrium (Figures 4B–4D). Therefore, the manubrium is not necessary to sustain satiety signals in the tentacles.

### Sustained satiety signals in the tentacles

**Figure 5:**
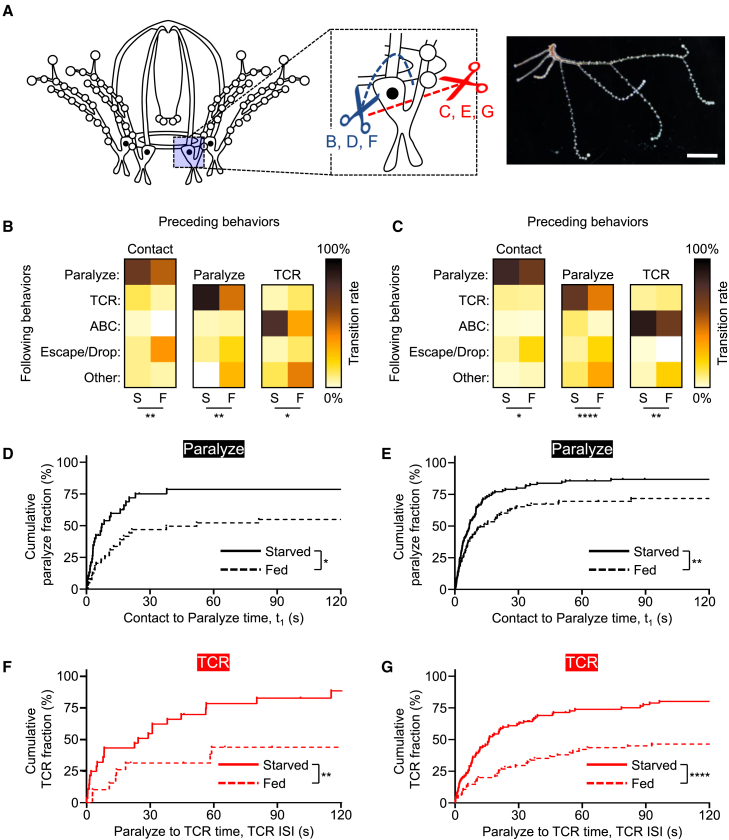
Sufficiency of satiety-sustaining signals in the tentacle (A) Jellyfish schematic indicating sites of tentacle dissection above (blue) and below (red) the tentacle bulb (left) and representative photograph of jellyfish tentacle dissected below the tentacle bulb (right). Scale bar, 1 mm. (B and C) Heat maps of transitional probabilities between feeding steps for tentacles from starved (S) and fed (F) jellyfish dissected above (B) and below (C) the tentacle bulb. From left to right, *n* = 40, 40, 28, 21, 24, and 9 (B) or *n* = 113, 108, 98, 76, 79, and 45 (C) observations per heatmap. Statistically significant differences from starved animals are indicated (Fisher’s exact test; ∗*p* < 0.05; ∗∗*p* < 0.01; ∗∗∗∗*p* < 0.0001). (D–G) Kaplan-Meier curves for cumulative fractions of paralyze (D and E) and TCR (F and G) for tentacles from starved (continuous) and fed (dashed) animals dissected above (D and F) and below (E and G) the tentacle bulb (log rank (Mantel-Cox) test; ∗*p* < 0.05; ∗∗*p* < 0.01; ∗∗∗∗*p* < 0.0001). From left to right, top to bottom, *n* = 40, 113, 28, 95 and 40, 108, 20, 75 observations for tentacles from starved and fed animals, respectively.

This striking finding (Figure 4) prompted us to examine the sufficiency of sustained satiety in tentacles separated from the umbrella. We severed tentacles both above and below the ocellus-containing tentacle bulb (Figure 5A), to test if this neuron-rich area plays a role in satiety. Isolated tentacles could execute feeding steps and were sufficient for detecting the effect of satiety on prey paralysis and TCR both with and without the tentacle bulb (Figures 5B–5G). Taken together, these surgical manipulations strongly suggest that satiety signals are transferred to and maintained in the tentacles after a meal. Given no differences with the tentacle bulb, the bulb neurons are also not necessary for maintaining the satiating effects in the tentacle.

### Distinct satiety dynamics for prey paralysis and the TCR

**Figure 6:**
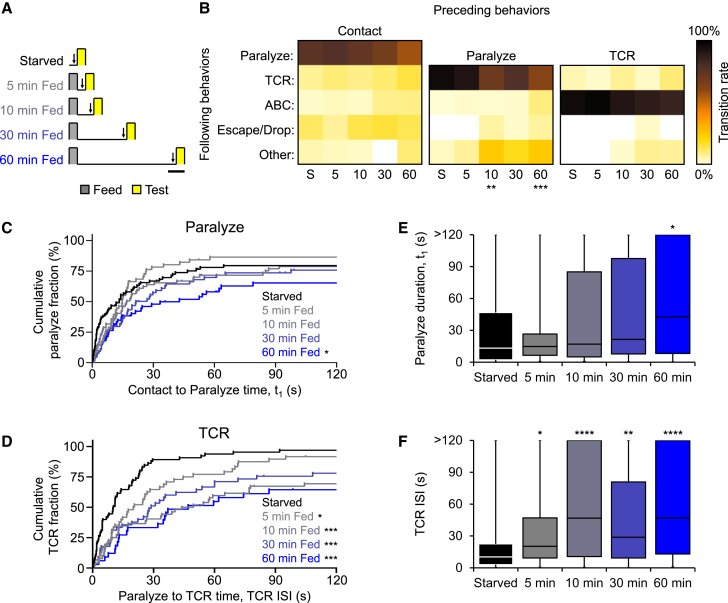
Rapid and distinct satiety dynamics for prey paralysis and the tentacle contraction reflex (TCR) (A) Feeding protocols tested for measuring satiety in isolated tentacles. Arrows indicate the time point of tentacle dissection. Scale bar, 10 min. (B) Heat maps of transitional probabilities between feeding steps for tentacles from starved jellyfish (S) and jellyfish after various intervals from feeding (5, 10, 30, 60). From left to right, *n* = 87, 62, 74, 67, 64, 66, 48, 52, 45, 34, 69, 48, 44, 41, and 32 observations per heatmap. Statistically significant differences from starved animals are indicated (chi-square test with Bonferroni corrections; ∗∗*p* = 0.0044; ∗∗∗*p* = 0.0008). (C and D) Kaplan-Meier curves for cumulative fractions of paralyze (C) and TCR (D) for tentacles derived from starved jellyfish (black) and jellyfish after various intervals from feeding (5, 10, 30, 60; gray to blue) (log rank (Mantel-Cox) test with Bonferroni corrections; ∗*p* < 0.05; ∗∗∗*p* < 0.001). From top to bottom, Starved to 60 min fed, *n* = 87, 62, 74, 67, 64, 66, 48, 52, 45, and 34 observations. (E and F) Effect of feeding on median paralyze duration (E) and TCR ISIs (F) (Kruskal-Wallis test; Dunn’s posttest; ns *p* > 0.05; ∗*p* < 0.05; ∗∗*p* < 0.01; ∗∗∗∗*p* < 0.0001). Lower, intermediate and upper bounds of boxes represent T_25_s, T_50_s, and T_75_s, respectively. Whiskers show minima/maxima. *n* = 79, 54, 66, 55, 50 (E) and *n* = 65, 47, 51, 44, 31 (F).

How quickly is satiety signaled to tentacles? To address this question, we offered animals a brief (5 min) meal and measured feeding behaviors of tentacles amputated at various time points after it (Figure 6A). Analysis revealed an overall tendency for reduction in feeding step probabilities as time from the meal increased (Figure 6B), gradually disorganizing the stereotyped feeding sequence. In particular, the dominant transition from prey paralysis to TCR observed in starved samples was significantly compromised in fed samples (Figure 6B). Interestingly, an increase in prey paralysis duration was statistically detectable only 1 h after feeding (Figures 6C and 6E). In contrast, inhibition of TCR was extremely rapid, occurring as quickly as 5 min after feeding and persisting until the end of the experiment (Figures 6D and 6F). Thus, satiety has strikingly distinct temporal dynamics on different steps of feeding behavior, likely mediated by different signals.

## Discussion

In cnidarians, ingestive feeding involves multiple steps. Studies with the freshwater polyp *Hydra* and anemones show that satiety regulates prey capture, tentacle responses, and mouth opening.^9^^,^^23^^,^^24^^,^^25^^,^^26^^,^^27^^,^^28^^,^^29^^,^^32^^,^^33^^,^^35^ In jellyfish, our previous work shows that prey paralysis duration and TCR onset are prolonged under satiety,^13^ but additional effects on the complete feeding sequence (Figure 1) are unknown. Here, we used the jellyfish *Cladonema* to address this question due to its unique combination of advantages. Unlike other jellyfish^31^ including the emerging model *Clytia*,^36^ *Cladonema* does not swim while feeding, and is thus amenable to precise quantification of this behavior. Moreover, its full feeding behavior is fast, proceeding from prey capture to ingestion typically within 2–3 min (Figure 2A). Since *Cladonema* feeding is a stereotyped sequence of clearly distinguishable steps (Figure 1), we could determine how satiety alters each step. We show that in *Cladonema*, satiety (1) inhibits and delays all steps of feeding (Figures 1 and 2), (2) occurs rapidly (Figure 6), and (3) is signaled throughout the body, allowing tentacles to autonomously inhibit feeding steps under their control (Figures 4 and 5).

In bilaterians, satiety suppresses feeding by raising sensory thresholds,^1^^,^^10^ but it is unclear if this also occurs in cnidarians. Feeding inhibits prey capture in *Cladonema* (Figures 1 and 2) and other cnidarians.^13^^,^^23^^,^^24^^,^^25^^,^^26^^,^^27^^,^^28^^,^^29^ Sting cell number reduction and/or their inhibition by satiety could explain this effect. Our results (Figure 3) and those of others in the sea anemone *Calliactis*^23^ argue against a role for sting cell reduction. In line with this, in *Hydra*, electric shock treatment to remove a subset of nematocytes did not alter animals’ ability to capture and ingest prey.^28^ Thus, inhibition of sting cell discharge rather than nematocyte reduction is the more likely underlying mechanism, and its primary purpose may be to conserve these single-use cells under satiety.

The effects of satiety in *Cladonema* were broad, inhibiting all feeding steps (Figures 1 and 2), which jointly contributed to decreasing food consumption in fed animals (Figure 1E). Interestingly, although fed jellyfish took longer to paralyze prey (Figures 2C and 2G), they still did so even when failing to ingest it (cf. plateaus in Figure 2C). Similar observations have been made for other cnidarians.^25^^,^^37^ Therefore, satiety modulation of downstream feeding steps is crucial for explaining reduced consumption in fed animals. Satiety especially prolonged the onset of TCR, increasing its median time more than 5-fold (Figure 2H). Moreover, the satiety effect on TCR onset was already detectable 5 min after feeding, while its effect on prey paralysis only reached significance after 1 h (Figures 6C–6F). These data highlight the importance of TCR in feeding suppression of fed animals. Independent regulation of prey paralysis and TCR in satiety (Figure 6) suggests different purposes to these steps: sting cell conservation and regulation of food intake, respectively.

*Cladonema* satiety signals are fast-acting and sustaining. Their sustained effects are consistent with peptidergic satiety molecules, arguing for a prevalence of peptidergic networks in cnidarians and perhaps their ancestors.^38^ This is in line with our previous findings, which show that the anorexigenic neuropeptide GLWamide phenocopies the satiety effect on TCR but not prey paralysis.^13^ Given no satiety effect on TCR duration (Figure 2K), GLWamide likely acts as a neuromodulator on motor neurons rather than directly controlling muscle contraction. As other peptides suppress food consumption in *Cladonema*^13^ and RFamide is involved in *Hydra* and *Clytia* feeding,^39^^,^^40^ it is reasonable to hypothesize that additional steps of feeding can be independently modulated.

What are the relationships between satiety molecules? In mammals, the hypothalamus is the master regulator of satiety, a function mediated by key neuropeptides it expresses.^41^ Hierarchical relationships between satiety molecules could exist in cnidarians. If so, it should be possible to identify “master regulator” molecules that control multiple aspects of feeding behavior. Alternatively, absence of stratified organization could be a general characteristic of the decentralized nervous systems of these animals. Thus, hierarchical networks could represent a later innovation in nervous system evolution that arose when stimulus integration became more complex. Determining which scenario holds true will offer unique insights into the evolution of nervous system centralization.

### Limitations of the study

Our study takes advantage of isolated jellyfish tentacles, as they can perform critical feeding steps. This enabled us to analyze the temporal dynamics of satiety without input from the manubrium. However, this reduced preparation only captures part of jellyfish feeding. For example, ingestion of captured prey requires the coordinated actions of the tentacles and manubrium. Feeding state-dependent modulation of this step is also important for food intake.

## Resource availability

### Lead contact

Requests for further information and resources should be directed to and will be fulfilled by the lead contact, Vladimiros Thoma (thoma.vladimiros.e3@tohoku.ac.jp).

### Materials availability

This study did not generate new unique reagents.

### Data and code availability

• Raw data from Figures 1, 2, 3, 4, 5, and 6 and Figure S1 were deposited on Mendeley Data: https://doi.org/10.17632/nndh863s5t.1.
• This paper does not report original code.
• Any additional information required to reanalyze the data reported in this paper is available from the lead contact upon request.

## Acknowledgments

This work was supported by MEXT/JSPS KAKENHI (20K15838 to V.T. and 19K22577, 22KK0106, and 24H01217 to H.T.) and the Tohoku University Research Program “Frontier Research in Duo” (to H.T.). Publication fees were covered by the Tohoku University FY2024 APC Support Program for Promoting Open Access.

## Author contributions

Conceptualization, V.T. and H. T.; methodology, G.M., and V.T.; formal analysis, G.M. and V.T.; investigation, G.M. and V.T.; writing – original draft, V.T.; writing – review and editing, V.T. and H.T.; visualization, G.M. and V.T.; supervision, V.T. and H.T.; funding acquisition, V.T. and H.T. V.T. dedicates this work to the memory of Thomas Thoma.

## Declaration of interests

The authors declare no competing interests.

## STAR★Methods

### Key resources table

**Table:** 

REAGENT or RESOURCE	SOURCE	IDENTIFIER
**Deposited data**		

Satiety differentially modulates feeding steps in the jellyfish *Cladonema*. Mashiba et al.	This work	Mendeley Data: https://doi.org/10.17632/nndh863s5t.1

**Experimental models: Organisms/strains**		

UN2 strain of *Cladonema pacificum*	Ryusaku Deguchi^13^	N/A

**Software and algorithms**		

GraphPad Prism 6	GraphPad, San Diego, CA	RRID: SCR_002798

**Other**		

*Artemia salina* eggs	Tetra, Japan	N/A
Artificial Sea Water	Gex, Japan	N/A

### Experimental model and study participant details

Adult jellyfish from the wild-type *Cladonema pacificum* male strain UN2^42^ were used for all experiments. Budding jellyfish (aged 0–1 day old) were collected from UN2 polyps, group housed and fed 5 days a week with an abundance of *Artemia salina* nauplii. After at least 1 h of feeding, jellyfish were transferred to fresh, filtered Artificial Sea Water (ASW). Jellyfish were maintained at 20°C in filtered ASW under a 12 h light:12 h dark cycle. Individual adult jellyfish, aged 3–5 weeks from budding, were used for behavioral experiments. Prior to most experiments (Figures 1, 2, 3, 4, and 5), jellyfish were allowed to feed *ad libitum* on an abundance of 1-day-old *Artemia salina* nauplii (Tetra, Japan) for 1 h. Under these conditions, jellyfish stopped feeding after approx. 20 min. After 1 h, their tentacles were covered with nauplii and their manubria were expanded, suggesting that they were fully satiated. Starved and fed jellyfish were tested 24–28 h or 0–1 h after feeding offset, respectively (unless otherwise noted). Research with *Cladonema* did not require ethical approval.

### Method details

Experiments were conducted in 24-well plates at 24°C under a Nikon SMZ745 microscope with dark-field illumination (Nikon P-DF LED Dark Field Unit). Prior to any treatment, jellyfish of the same age appeared identical, and were thus randomly assigned to control or experimental groups. However, due to the expanded manubrium of freshly fed jellyfish, the experimenter could not be blinded to animal feeding state. Videos of feeding behavior were recorded with a Nikon 1 J4 camera and manually annotated. Typically, the time points of contact with the prey, completion of prey paralysis and the onsets/offsets of the TCR, tentacle bending and prey ingestion were determined, where available. The numbers of replicates per experiment are as follows: Figures 1, 2 and S1: 6; Figure 3: 4; Figure 4: 2; Figures 5B, 5D and 5F: 11; Figures 5C, 5E and 5G: 23; Figure 6: 25. Sample sizes were selected to conform to field standards and our previous work.^13^

#### Intact jellyfish behavior

Each jellyfish was placed in a well with 1 mL of ASW and sequentially offered 5 individual 1-day-old brine shrimps. Following contact with the prey, 2 min were offered for observation of each step of feeding behavior.

#### Feeding experiments with tentacle targeting

To provide food to specific jellyfish tentacles (Figure 3), brine shrimps were anesthetized with brief exposure to CO_2_. An excess of anesthetized shrimps was applied on 4 *Cladonema* tentacles. Non-captured shrimps were immediately removed. The process was repeated thrice. No shrimps were applied to ‘unused’ tentacles. Naturally occurring markers such as branched or connected radial channels^43^ were used to distinguish tentacles. Following 1 h from feeding onset, jellyfish were offered more than 10 free-swimming (non-anesthetized) shrimps and videos of their behavior were recorded.

#### Resection experiments

Individuals were pre-treated as intact jellyfish above. For manubrium (Figure 4) and tentacle (Figure 5) resections, fine dissection scissors were used to cut the umbrella near the base of the manubrium or the tentacle above or below the ocellus, respectively. Preparations were tested in well plates (one manubrium-less jellyfish or three tentacles per well). For severed tentacles, only one brine shrimp was used per well.

#### Satiety dynamics measurements

To determine satiety dynamics (Figure 6), animals were starved or allowed to briefly feed *ad libitum* with an abundance of shrimp nauplii for 5 min. Tentacles were severed below the ocellus and washed directly prior to tests. Measurements were carried out with severed tentacles after 24–28 h starvation or after 5, 10, 30 or 60 min from the offset of the brief (5 min) feeding as described above (resection experiments).

### Quantification and statistical analysis

All statistical tests were carried out with Prism 6 (GraphPad, San Diego, CA). Nonparametric tests were chosen, as they are more robust. Behavioral data were analyzed for various parameters to determine how satiety alters feeding. To visualize feeding step transitions, heat maps were generated and the underlying data compared with Fisher’s exact test or a chi-square test (Figures 1C, 1F, 3B, 4B, 5B, 5C and 6B). To analyze feeding step timing and success, Kaplan-Meier plots were generated and compared with log rank (Mantel-Cox) tests with Bonferroni corrections (Figures 2C–2F, 3C, 4C, 4D, 5D–5G and 6C, 6D). In some cases (Figures 2G–2J, 3D, 6E, 6F), these data were also summarized as box-and-whisker plots, with lower, intermediate and upper box bounds representing T_25_s, T_50_s and T_75_s, respectively, and whiskers showing minima/maxima. Underlying uncensored data were compared with Mann-Whitney tests with Bonferroni corrections (Figures 2G–2J, 3D) or Kruskal-Wallis tests with Dunn’s posttest (Figures 6E and 6F). TCR, Bending and Ingestion durations are shown as box-and-whisker plots, with lower, intermediate and upper box bounds representing the first, second and third quartiles, respectively, and whiskers showing minima/maxima; underlying data were compared with Mann-Whitney tests (Figures 2K–2M). Total shrimp consumption is shown as average ±s.e.m. and compared with a Mann-Whitney test (Figure 1E). All tests were two-tailed, where applicable. Significance levels are as follows: ns *p* > 0.05; ∗*p* < 0.05; ∗∗*p* < 0.01; ∗∗∗*p* < 0.001; ∗∗∗∗*p* < 0.0001. Statistical details for all experiments are provided in the figure legends.
