# Supplementary material for: Satiety differentially modulates feeding steps in the jellyfish Cladonema
Source: iScience. 2025 Mar 10;28(4):112192. doi: 10.1016/j.isci.2025.112192 (PMC11985073; doi:10.1016/j.isci.2025.112192)
Supplement: Document S1. Figure S1 [file mmc1.pdf]

iScience, Volume *28*

Supplemental information

Satiety differentially modulates feeding steps in the jellyfish *Cladonema*

Genta Mashiba, Hiromu Tanimoto, and Vladimiros Thoma

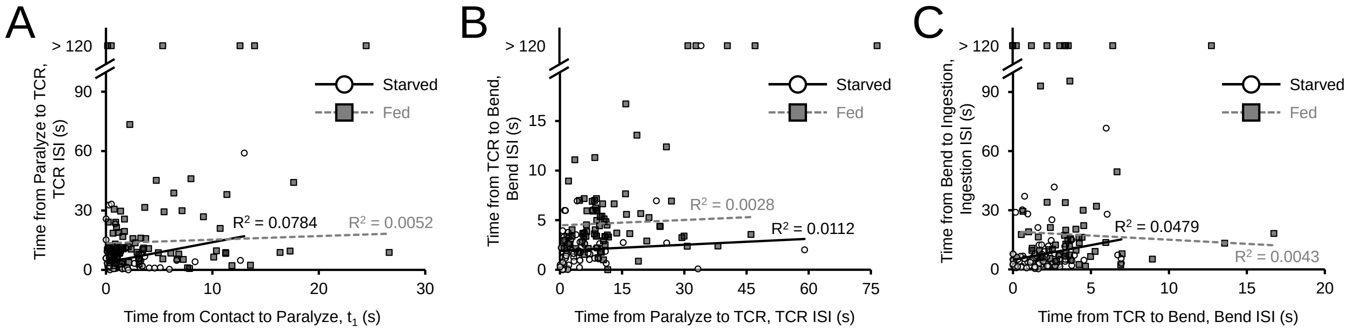

Figure S1: Timing of adjacent feeding steps is not correlated. (A-C) Correlations between times from prey contact to prey paralysis (t_1) and prey paralysis to Tentacle Contraction Reflex onset (TCR ISI) (A); between TCR ISI and TCR offset to Bend onset (Bend ISI) (B); between Bend ISI and Bend Offset to Ingestion onset (Ingestion ISI) (C). Data and their linear regressions from starved (white circles, solid black line) and fed (gray boxes, dashed gray line) animals are shown alongside R-squared values. From left to right, *n* = 78, 78, 86 and 86, 78, 60 for starved and fed animals, respectively.
